# Structured on-the-job training to improve retention of newborn resuscitation skills: a national cohort Helping Babies Breathe study in Tanzania

**DOI:** 10.1186/s12887-019-1419-5

**Published:** 2019-02-07

**Authors:** Mary Drake, Dunstan R. Bishanga, Akwila Temu, Mustafa Njozi, Erica Thomas, Victor Mponzi, Lauren Arlington, Georgina Msemo, Mary Azayo, Allan Kairuki, Amunga R. Meda, Kahabi G. Isangula, Brett D. Nelson

**Affiliations:** 1 Jhpiego, Plot 72, Block 458, New Bagamoyo Road, Victoria, Dar es Salaam, Tanzania; 2 Division of Global Health, Department of Pediatrics, Massachusetts General Hospital, 125 Nashua Street, 8th Floor, Boston, MA 02114 USA; 3 Ministry of Health and Social Welfare, 36/37 Samora Avenue, Dar es Salaam, Tanzania; 4 Harvard Medical School, 25 Shattuck Street, Boston, MA 02115 USA; 5 University of Groningen, University Medical Centre Groningen, Department of Health Sciences, GlobalHealth, Groningen, the Netherlands

**Keywords:** Newborn resuscitation, Birth asphyxia, Newborn health, Skills retention, On-the-job training, Helping babies breathe, Tanzania, Low-income countries

## Abstract

**Background:**

Newborn resuscitation is a life-saving intervention for birth asphyxia, a leading cause of neonatal mortality. Improving provider newborn resuscitation skills is critical for delivering quality care, but the retention of these skills has been a challenge. Tanzania implemented a national newborn resuscitation using the Helping Babies Breathe (HBB) training program to help address this problem. Our objective was to evaluate the effectiveness of two training approaches to newborn resuscitation skills retention implemented across 16 regions of Tanzania.

**Methods:**

An initial training approach implemented included verbal instructions for participating providers to replicate the training back at their service delivery site to others who were not trained. After a noted drop in skills, the program developed structured on-the-job training guidance and included this in the training. The approaches were implemented sequentially in 8 regions each with nurses/ midwives, other clinicians and medical attendants who had not received HBB training before. Newborn resuscitation skills were assessed immediately after training and 4–6 weeks after training using a validated objective structured clinical examination, and retention, measured through degree of skills drop, was compared between the two training approaches.

**Results:**

Eight thousand, three hundred and ninety-one providers were trained and assessed: 3592 underwent the initial training approach and 4799 underwent the modified approach. Immediately post-training, average skills scores were similar between initial and modified training groups: 80.5 and 81.3%, respectively (*p*-value 0.07). Both groups experienced statistically significant drops in newborn resuscitation skills over time. However, the modified training approach was associated with significantly higher skills scores 4–6 weeks post training: 77.6% among the modified training approach versus 70.7% among the initial training approach (*p*-value < 0.0001). Medical attendant cadre showed the greatest skills retention.

**Conclusions:**

A modified training approach consisting of structured OJT, guidance and tools improved newborn resuscitation skills retention among health care providers. The study results give evidence for including on-site training as part of efforts to improve provider performance and strengthen quality of care.

**Electronic supplementary material:**

The online version of this article (10.1186/s12887-019-1419-5) contains supplementary material, which is available to authorized users.

## Background

The world has made remarkable progress over the past two decades in reducing child mortality, however, the pace was insufficient to universally achieve Millennium Development Goal 4, or the reduction of under-five mortality by two-thirds. Only about a third of countries, including Tanzania, was able to achieve this target [1]. The decline in neonatal mortality has been slower than that of post-neonatal under-five mortality and has only dropped from 21 per 1000 to 20 per 1000 [2]. To achieve the Sustainable Development Goal of reducing neonatal mortality to 12 per 1000 live births or lower, we must end preventable deaths. Birth asphyxia remains the leading cause of newborn mortality and accounts for nearly 900,000 neonatal deaths annually [3].

Strengthening the capacity of health workers to provide newborn resuscitation is critical to reducing newborn deaths. In response to this challenge, the American Academy of Paediatrics and other partners developed the Helping Babies Breathe (HBB) curriculum [4] to teach providers how to respond to asphyxiated newborns with life-saving measures. The HBB course utilizes simulation like many other life-saving skills courses [5].

In addition to acquiring initial competency, retention of skills over time is extremely important. Retention of knowledge and clinical skills following training, however, has been reported as a challenge in most settings. In a systematic review of literature on newborn resuscitation trainings, up to 50% of studies analysed demonstrated significant performance decline over time [6]. To improve birth asphyxia outcomes, studies of health care worker knowledge and skills following newborn resuscitation training indicate increased emphasis should be placed on health care workers’ self-efficacy and mastery of newborn resuscitation skills [7]. It has been shown in similar studies that skills drops could happen as early as 12 weeks following training, suggesting that stand-alone training, without follow-up activities for trainees to practice learned skills, may not be adequate in resource-limited settings [8, 9]. Some training programmes with no drop in skills were characterized by having structured follow-up activities to reinforce materials learned at primary training such as refresher trainings and mentoring [6].

Tanzania has substantially reduced child mortality in the past 10 years, but most of the decline has come after the first month of life with neonatal mortality remaining largely unchanged. Each year, at least 51,000 Tanzanian newborns die, and an additional 43,000 babies are stillborn [10]. The neonatal mortality rate in Tanzania remains high at 21 deaths per 1000 live births, with birth asphyxia being one of the leading causes, contributing to 31% of deaths [11]. A 2009–2011 pilot study of HBB within eight healthcare facilities in Tanzania produced a 47% reduction in facility-based early neonatal mortality rate within 24 h of life [12]. These results prompted the Tanzania Ministry of Health (MoH) to prioritize implementing HBB nationwide with support from the Children’s Investment Fund Foundation (London, UK).

The MoH and Jhpiego worked to rapidly implement a 3-year, capacity-building programme in newborn resuscitation. Programme data to measure providers’ retention of HBB skills was analysed to assess whether modifications to the training approach could enhance retention of newborn resuscitation skills in Tanzania.

## Methods

### HBB Programme implementation

Between May 2013 and March 2015, a national HBB training programme was implemented across Tanzania within all public and faith-based health facilities conducting deliveries in a phased region-by-region approach across 16 of Tanzania’s 26 mainland regions [13]. The training targeted all health cadres working in labour and delivery rooms and obstetric theatres. From May 2013 until March 2014, an initial training approach was used in eight regions (Pwani, Lindi, Dar, Morogoro, Iringa, Njombe, Ruvuma, Mbeya) reaching 6724 providers. From May 2014 to March 2015, the modified training approach was used in eight new regions (Manyara, Arusha, Kilimanjaro, Tanga, Singida, Kigoma, Kagera, Mara) and reached 6480 providers.

### Initial training approach

The HBB course in Tanzania followed the Helping Babies Breathe curriculum (first edition) developed by the American Academy of Pediatrics (AAP) and its partners, the training materials were adapted to fit the Tanzanian context and translated into Kiswahili. The main topics of the HBB course focused on providing knowledge and skills in preparation for birth, routine newborn care, ensuring breathing or ventilation by the Golden Minute, and improving ventilation. The training objectives were achieved through adult-learning approaches and frequent hands-on practice with the NeoNatalie newborn mannequin (Laerdal Global Health, Stavanger, Norway).

The training was implemented in a cascade approach, with national trainers working with district-level trainers to train facility-based health care providers. Trainings were held at convenient centralized locations such as primary schools and hotel conference rooms. Each training was led by a national trainer and several district trainers and targeted 20 health care providers per hospital, eight per health centre, and three per dispensary. The 1-day trainings maintained a trainer-trainee ratio of 1:6 and a trainee-mannequin ratio for skills practice of 2:1 or 3:1. An immediate post-training assessment using a previously validated skills checklist, or objective structured clinical exam (OSCE) tool [14], was conducted. In place of the AAP’s two separate HBB OSCEs A and B, the MoH chose to utilize this single-scenario OSCE to streamline implementation and longitudinal evaluation of this at-scale HBB program in a resource-limited setting.

Upon completion of the training, providers were given HBB equipment and training materials for their facilities, including a NeoNatalie mannequin, HBB Learner’s Guide, HBB Action Plan poster, and HBB clinical reminder wall poster [4]. Initially, HBB-trained providers were instructed and encouraged during training to use these materials to practice their skills and conduct on-the-job-training for peer-to-peer continuous learning among trained providers as well as to train any labour ward staff who had not attended the centralized training, including newly assigned staff [13]. Providers promised to conduct the training back at their facilities and to keep practicing their own HBB skills. Four to 6 weeks following the training, a national HBB trainer and a district HBB trainer conducted joint follow-up visits with the trained HBB providers at each facility. During these visits, the trainers assessed whether the facility had a dedicated space for newborn resuscitation, HBB-trained providers in the labour ward, and the provided newborn resuscitation supplies and equipment. The same OSCE used during immediate post-training assessment was repeated; the trainers also assessed skills of both the providers who attended the HBB training session and those who received on-the-job training. Providers who did not perform well on the OSCE were re-instructed until they could perform newborn resuscitation according to HBB guidelines.

### Modified training approach

During the first annual HBB programme review in February 2014, results from the skills assessments immediately after initial training and 4–6 weeks after training were reviewed. The programme implementation team noted with concern that OSCE scores at the 4–6-week follow-up visits were considerably lower than scores immediately post-training. Additionally, the programme found that, when trained providers returned to their facilities, there was limited self-initiated practice [13]. In follow-up visits, supervisors learned providers had typically only provided their colleagues with a verbal report of what had transpired in the training, and in many facilities, the newborn mannequin was found unused, still in its packaging. It was hypothesized that the drop in skills since training was due to providers not sufficiently practicing their HBB skills after returning to their facilities.

In response to these findings, the programme introduced a structured on-the-job training (OJT) tool (Additional file 1) to facilitate self-learning as well as peer-to-peer continuous learning via hands-on HBB practice scenarios with the NeoNatalie mannequin. A team of local and international newborn health specialists led by the Tanzania MoH HBB master trainers worked together between March – May 2014 to design, pre-test, and develop the Swahili-language HBB OJT tool (S1). The tool is a 13-page, simple, user-friendly guide designed for group practice of four critical HBB skill areas: preparation for birth, routine newborn care, ensuring ventilation within the Golden Minute, and improving ventilation when needed. The guide provides instruction on organizing and conducting individual or group practice using the NeoNatalie mannequin limited to two patient scenarios. These two patient scenarios included 1) preparation for birth with routine initial newborn care (Scenario 1) and 2) resuscitation within the Golden Minute and improving inadequate ventilation (Scenario 2). The structured OJT approach was introduced by orienting district trainers through a refresher training. District trainers then included the approach in subsequent provider trainings at the end of the 1-day HBB training agenda, where participants were asked to indefinitely continue the OJT for their own self-learning on HBB skills as well as for peer training. This structured guidance on conducting OJT was then integrated into all subsequent trainings beginning in May 2014. The OJT was led by a HBB champion (usually also a supervisor of the maternity or RCH services), who was trained an additional day beyond the 1-day HBB training on effective clinical teaching skills, how to organize the sessions at the site, and identification of skills gaps.

#### Implementation and monitoring of OJT

Implementation: OJT Champions (in most cases in-charge of the labour ward) coordinated implementation and recording of OJT activities. To identify clinical skills gaps, the champions reviewed the labor and delivery registers to triangulate APGAR scores, HBB steps applied, and neonatal status at discharge (alive or deceased). Discrepancies in the documentation by a certain provider led to that provider being prioritized. The champion would also note clinical skills gaps by observing delivery room care. The skills gaps identified then drove the practice to be focused on the specific skills needed, rather than doing a longer series of skills. In general, providers would practice a skill for 10–15 min.

Monitoring: During the 4–6-week follow-up visits, the assessors would review the OJT through documentation of all facility-based OJT plans and activities in the designated register placed in the labor ward.

### Data collection and analysis

#### Knowledge and skills assessment

At the conclusion of each training, trainers assessed each provider’s HBB knowledge and skills using the OSCE that was previously developed for the Tanzanian HBB programme. A national or district trainer administered the OSCE to each provider individually using the NeoNatalie newborn mannequin. The assessment went through a single patient scenario, during which the provider used a mannequin to demonstrate competence in preparing for birth, drying thoroughly, bulb suctioning and tactile stimulation, ventilating with a bag-mask device, and improving inadequate ventilation. (Of important note, this study was conducted prior to the second edition of the HBB training program, in which bulb suctioning is now recommended only in the event of suspected airway obstruction [15]. Each of the OSCE’s 13 items were considered essential neonatal resuscitation skills, and points were awarded to providers for each successfully completed item. The items considered of greatest importance scored three points each, whereas those of lesser importance scored one point each. Total scores could range from 0 to 23. Providers who achieved scores of at least 16 were rated in the “green” category, meaning that no further clinical coaching was deemed necessary before they could provide HBB services, but they were encouraged to do continuous practice back at their facility to retain the skills. Providers scoring 15 or lower (“red” category) were given further on-site clinical coaching.

Data from the few trainees who do not provide direct clinical care – such as hospital administrators, lab technicians, etc. – were removed from the analysis. Data from trainees for whom there were not OSCE scores from both immediately after training and 4–6 weeks after training were also excluded. To help assess for differences in skills acquisition and retention across provider training and clinical experience, healthcare providers were categorized into three groups: nurses/midwives, medical attendants (semi-trained healthcare works often at lower-level health facilities), and other clinicians (medical officers, assistant medical officers and clinical officers).

Data analysis was conducted using Stata 14 (College Station, Texas). Comparisons of cadre distributions were done using *χ*^2^. Skills retention, measured through degree of skills drop, was compared over time (baseline versus 4–6 weeks post-training) and between the two training approaches using *t*-tests. This study was approved by the institutional review board of Partners HealthCare (Massachusetts General Hospital, Boston, MA, USA), the National Institute for Medical Research (Dar es Salaam, Tanzania), and the MoH of Tanzania (Dar es Salaam, Tanzania).

## Results

### Distribution of trained health care providers

Paired data from immediately post-training and 4–6 weeks after training were available from 8391 trainees and were included in the analysis – 3592 undergoing the initial training approach (May 2013–March 2014) and 4799 undergoing the modified approach (May 2014–March 2015) (Table 1). Of these 8391 health care providers, half were nurses/midwives (50.0%), followed by medical attendants (33.0%) and, lastly, other clinicians (17.0%). There was a higher proportion of nurses / midwives in the modified training compared to the initial training and a higher proportion of medical attendants during the initial training approach compared to the modified training approach.

**Table 1:** Comparison of cadre distribution by training approach^a^

Cadre	Initial Approach	Modified Approach	Total
	n (%)	n (%)	n (%)
Nurses / midwives	1682 (46.8)	2516 (52.4)	4198 (50.0)
Medical attendants	1279 (35.6)	1492 (31.1)	2771 (33.0)
Other clinicians	631 (17.6)	791 (16.5)	1422 (17.0)
Total	3592	4799	8391

^a^Data from participants for which we have data on the same individual at immediate post-training and at 4–6 weeks follow-up

### Comparison of baseline OSCE scores

When looking at all cadres combined, there was no statistically significant difference in OSCE results immediately after training between the two training approaches, with mean scores of 80.5 and 81.3% for initial and modified training, respectively (Table 2).

**Table 2:** Mean OSCE scores and standard deviations in initial and modified training groups – immediately after training and 4–6 weeks after training

	Initial training group			Modified training group			*P*-value^***^
	Immediately post-training	4–6 weeks post-training	*P*-value^*^	Immediately post-training	4–6 weeks post-training	*P*-value^**^	
Overall scores							
All	80.5 (79.8–81.2)	70.7 (69.9–71.5)	< 0.01	81.3 (80.7–81.9)	77.6 (77.0–78.3)	< 0.01	0.07
Nurses / midwives	82.7 (81.7–83.7)	74.9 (73.8–76.0)	< 0.01	84.8 (84.0–85.5)	80.7 (79.8–81.5)	< 0.01	< 0.01
Medical attendants	76.6 (75.4–77.9)	65.1 (63.7–66.5)	< 0.01	74.2 (73.0–75.3)	72.5 (71.3–73.8)	0.02	< 0.01
Other clinicians	82.5 (80.9–84.1)	70.7 (68.7–72.7)	< 0.01	83.9 (82.5–85.2)	77.7 (76.0–79.3)	< 0.01	0.19
Suctioning							
All	86.8 (85.7–87.9)	75.4 (74.0–76.8)	< 0.01	89.4 (88.5–90.3)	84.9 (83.9–85.9)	< 0.01	< 0.01
Nurses / midwives	88.8 (87.3–90.3)	80.2 (78.3–82.0)	< 0.01	91.8 (90.7–92.9)	87.5 (86.2–88.8)	< 0.01	< 0.01
Medical attendants	84.3 (82.3–86.3)	70.1 (67.7–72.6)	< 0.01	84.5 (82.7–86.4)	81.3 (79.3–83.3)	< 0.01	0.87
Other clinicians	86.8 (84.2–89.5)	73.4 (70.0–76.8)	< 0.01	91.0 (89.0–93.0)	83.4 (80.8–86.0)	< 0.01	0.01
Stimulation							
All	66.0 (64.4–67.5)	51.8 (50.1–53.4)	< 0.01	70.3 (69.0–71.6)	60.0 (58.6–71.6)	< 0.01	< 0.01
Nurses / midwives	68.3 (66.0–70.5)	56.7 (54.3–59.1)	< 0.01	74.5 (72.8–76.2)	64.8 (63.0–66.7)	< 0.01	< 0.01
Medical attendants	61.1 (58.4–63.7)	44.7 (42.0–47.5)	< 0.01	60.7 (58.2–63.2)	52.5 (50.0–55.1)	< 0.01	0.86
Other clinicians	69.7 (66.1–73.3)	52.9 (49.0–56.8)	< 0.01	74.7 (71.7–77.8)	58.9 (55.5–62.3)	< 0.01	0.04
Bag-mask ventilation							
All	85.4 (84.6–86.3)	79.2 (78.2–80.2)	< 0.01	83.1 (82.4–83.9)	83.8 (83.1–84.6)	0.91	< 0.01
Nurses / midwives	87.7 (86.6–88.8)	82.7 (81.4–84.0)	< 0.01	86.9 (85.8–87.6)	86.1 (85.0–87.1)	0.17	0.15
Medical attendants	81.4 (79.9–82.9)	74.3 (72.4–76.1)	< 0.01	76.1 (74.5–77.6)	79.3 (77.7–80.8)	1.00	< 0.01
Other clinicians	87.5 (85.7–89.3)	79.7 (77.3–82.2)	< 0.01	85.1 (83.3–86.8)	85.5 (83.6–87.3)	0.62	0.06

^*^P-value: Comparison of scores immediately post-training and 4–6 weeks post-training among initial training group

^**^P-value: Comparison of scores immediately post-training and 4–6 weeks post-training among modified training group

^***^P-value: Comparison of immediately post-training scores among the initial training and modified training groups

The baseline scores were higher for midwives in the modified group, while the baseline scores for medical attendants were lower in the modified group. There was no significant difference in baseline scores for the other clinician group. Statistically higher baseline scores were observed for stimulation and bulb suction in the modified training approach, while scores for bag-mask ventilation were lower. In the modified training group, bag-mask ventilation skills were significantly lower only among medical attendants, while nurses/midwives and the other clinicians group had higher baseline scores in bulb suctioning and tactile stimulation.

### Assessment of skills drop within the initial training approach

For those trained with the initial training approach, mean scores on the 4–6 weeks follow-up assessment were lower than the mean scores on the immediate post-training assessment, with the overall mean score going from 80.5 to 70.7% (Table 2). The skills drop was statistically significant in each of the three cadre categories and for each of the HBB steps. By cadre, nurses/midwives had higher skills retention as compared to medical attendants and other clinicians. By skill, the highest scores were seen for bag-mask ventilation and bulb suctioning, while scores for tactile stimulation of the non-vigorous newborn were much lower.

### Assessment of skills drop within the modified training approach

With the modified training approach, there was a statistically significant decrease in scores from immediately after training to 4–6 weeks post-training (Table 2). When observing skills drop by cadre, medical attendants had greater retention of skills, whereas the category of other clinicians had the largest drop. Among the medical attendant cadre, the skills drop varied by skill. For suctioning and stimulation, there was a significant drop, however, for bag-mask ventilation, the scores were slightly higher at 4–6 weeks follow-up.

### Comparison of skills drop by training approach

There were statistically significant declines in skills in both training approaches, but the decline among providers who participated in the modified training approach was significantly less than those who participated in the initial training approach (Table 3). Medical attendants had greater success in the modified training approach, preventing up to 10 points in skills drop compared to the initial approach. Among all cadres, the average skills drop went from 14.2 points in the initial training approach to 10.2 points in the modified training approach. Notably, with the modified training approach, there was no significant fall off in bag-mask ventilation skills. Retention of tactile stimulation skills showed less improvement with the modified approach compared to retention of bulb suction and bag-mask ventilation skills.

**Table 3:** Comparison of differences in skills drop between the initial approach and the modified approach

Cadre	Initial Training skills drop	Modified Training skills drop	*P* value
Overall HBB OSCE			
All	9.8 (8.9–10.8)	3.7 (2.9–4.5)	< 0.01
Nurses / midwives	7.8 (6.4–9.1)	4.1 (3.1–5.1)	< 0.01
Medical attendants	11.6 (9.9–13.2)	1.7 (0.0–3.3)	< 0.01
Other clinicians	11.8 (9.5–14.1)	6.2 (4.3–8.1)	< 0.01
Suction			
All	11.4 (9.7–13.2)	4.5 (3.2–5.8)	< 0.01
Nurses / midwives	8.6 (6.2–11.0)	4.3 (2.7–6.0)	< 0.01
Medical attendant	14.2 (11.0–17.3)	3.2 (0.5–5.9)	< 0.01
Other clinicians	13.5 (9.4–17.6)	7.6 (4.4–10.7)	0.02
Stimulation			
All	14.2 (12.1–16.2)	10.2 (8.5–12.0)	< 0.01
Nurses / midwives	11.5 (8.6–14.5)	9.7 (7.4–12.0)	0.17
Medical attendant	16.3 (12.9–19.8)	8.2 (4.8–11.6)	< 0.01
Other clinicians	16.8 (12.1–21.7)	15.8 (11.7–20.0)	0.38
Bag-mask ventilation			
All	6.3 (5.0–7.5)	−0.7 (− 1.8–0.3)	< 0.01
Nurses / midwives	5.0 (3.4–6.7)	0.6 (− 0.7–2.0)	< 0.01
Medical attendant	7.1 (4.9–9.4)	− 3.2 (− 5.3 - -1.1)	< 0.01
Other clinicians	7.8 (4.9–10.6)	−0.4 (− 2.9–2.1)	< 0.01

## Discussion

Participants trained in the initial and modified training approaches displayed similar acquisition of HBB skills, as shown by statistically equivalent scores immediately after training. Nurses/midwives and the cadre of other clinicians obtained higher scores compared to those of medical attendants. Skills were also generally higher for bulb suctioning and bag-mask ventilation and lower for tactile stimulation of the non-vigorous newborn.

In assessments conducted 4–6 weeks after training, skills drops were observed among participants in both training approaches, and this drop occurred in all three critical skills: bulb suctioning, stimulation, and bag-mask ventilation. Similar findings of fall off in skills over time have been seen in other studies [6, 15]. Some studies have indicated that skills drops could happen as early as within 3 months following structured resuscitation trainings [5, 7, 16]. Findings from this study add to evidence specifically from low- and middle-income countries that a significant fall off in skills can be identified even earlier after initial training -- within 4–6 weeks in both training approaches. A study from India and Kenya by Bang et al. [17] reported on factors associated with loss of skills post-initial training to include providers from higher-level health facilities, having no prior resuscitation training, and timing of training. Of these factors, lack of prior training in newborn resuscitation could apply to our study since this was the first time HBB was being rolled out in intervention health facilities.

However, this natural fall off in provider-level skills can be effectively addressed. Follow-up OSCE scores in the modified training approach show improved skills retention among every healthcare cadre, with the most dramatic attenuation in skills drop occurring among medical attendants who were expected to have had less exposure to newborn resuscitation skills based on their regular roles compared to clinicians and midwives. This kind of improved performance across cadres after HBB training was reported in another study in Honduras [18].

A systematic review showed that simulation and refresher training improve skills retention [6]. This reveals that there are ways of making skills last longer with modification in training approaches. We hypothesize that the reduced skills drop in the modified training group was mainly due to the introduction of a modified training approach which included structured OJT tools and supervision. Another study in Honduras showed similar improved retention of skills from monthly practice [19]. A study from India and Kenya demonstrated that providers could retain almost universal knowledge but not skills following an initial HBB training [17], whereas providers could retain bag-and-mask skills 6 months after training by instituting follow on activities in Nepal [20]. Accordingly, the structured guide was designed to reinforce self-directed, repetitive practice of HBB skills at the workplace. The OJT included clearly defined learning outcomes and directed providers to have regular practice of skills through simulation, which are other attributes that have been reported to facilitate learning [5]. These factors were absent from the design of the initial training approach, which despite providing instructions for participants to continue practicing with simulation, lacked any structured tool to reinforce that behaviour. Our findings also support the importance of workplace-based practice, consistent with other published work [6, 21] as well as repeated exposure to learning interventions to improve knowledge and skills retention [22].

Previous studies have noted the challenges of linking skills demonstration with improved practice and clinical outcomes [6, 9, 23]. Promisingly, some recent studies in Ghana show linkages between modified training approaches such as low-dose high frequency (LDH) and workplace-based simulation for improved skills retention and improved clinical outcomes including newborn death within 24 h and intrapartum stillbirth [15, 20, 24, 25]. Such interventions are appropriate to low-resource settings being reported to be cost effective and presenting value for money [26, 27]. The modified training approach that we studied has similar characteristics to those studies including the repetitive exposure to training, use of simulation, and being conducted at the workplace. Similar characteristics are emphasized in the second edition of HBB that reinforces learning through practice, fosters facilitator mentoring, and promotes quality improvement [28]. Some elements that differ may represent missing pieces of the puzzle to ensure training programs that lead to better outcomes, such as training in a package of care rather than a single condition, and utilizing SMS for mentoring and making sustainability a core feature of the training approach [29]. Additionally, strengthening timeliness, completeness and accuracy of data through training, supervision and mentorship is a critical element to better study the link between clinical training, application of practices and clinical outcomes.

### Limitations

Inclusion of many participants from different regions is a major strength of this study but also may be a limitation. This is because the current study is based on the country-level implementation of HBB in Tanzania where health service organization and challenges may be different from other countries even within a similar income group. Therefore, the results should be considered in the context of a low-resource setting where large-scale training on newborn resuscitation had not previously been established. Since the initial and modified training approaches were implemented sequentially, the results may have been impacted by any interim events, however, we are not aware of any interim trainings or other potential confounders. Additionally, this study did not look at clinical outcomes due to data limitations (availability of timely, complete and accurate data). Lastly, data on skills retention are limited in this study to the follow-up period of 4–6 weeks.

## Conclusion

This study demonstrates that retention of HBB skills can be significantly improved by including the contextually structured OJT tools in HBB program. This was particularly evident among medical attendants, who in many low- and middle-income countries are the only healthcare cadre present in lower-level health facilities. It will be useful to have study on further mastery of skills as well as retention beyond 4–6 weeks after training. Furthermore, additional studies to identify the optimal package of OJT such as which competencies to practice, for what duration, at what frequency, timing during the work day, mix of provider types to do OJT together, user-friendly tracking and reporting tools, as well as feasibility of implementing OJT as a routine approach can contribute to further increasing skills retention of providers performing this life-saving intervention.

## Additional file

Additional file 1: BMC Pediatrics Supplementary File. Supplemental Material 1: On-the-job Training Tool. Description:On-the-job training tool to improve retention of Helping Babies Breathe skills. (PDF 283 kb)

## Abbreviations

AAP: American Academy of Pediatrics
HBB: Helping babies breathe
MoH: Ministry of Health
OJT: On-the-job training
OSCE: Objective structured clinical exam
