# Supplementary material for: Structured on-the-job training to improve retention of newborn resuscitation skills: a national cohort Helping Babies Breathe study in Tanzania
Source: BMC Pediatr. 2019 Feb 7;19:51. doi: 10.1186/s12887-019-1419-5 (PMC6366017; doi:10.1186/s12887-019-1419-5)
Supplement: Supplementary file 1 — BMC Pediatrics Supplementary File. Supplemental Material 1: On-the-job Training Tool. Description:On-the-job training tool to improve retention of Helping Babies Breathe skills. (PDF 283 kb) [file 12887_2019_1419_MOESM1_ESM.pdf]

Supplemental Material 1: On-the-job Training Tool

JAMHURI YA MUUNGANO WA TANZANIA

WIZARA YA AFYA NA USTAWI WA JAMII

(Ministry of Health and Social Welfare Tanzania)

MWONGOZO WA MAFUNZO KAZINI KWA WATOA HUDUMA KUHUSU JINSI YA KUMSAIDIA MTOTO MCHANGA KUPUMUA

(On-the-job training guide for health care workers on Helping Babies Breathe)

AFYA YA UZAZI NA MTOTO, APRILI, 2014

REPRODUCTIVE AND CHILD HEALTH, APRIL 2014

UTANGULIZI ***(INTRODUCTION)***

Mafunzo ya jinsi ya kumsaidia mtoto mchanga kupumua ni muhimu sana kwa kila mtoa huduma anayemhudumia mama mjamzito wakati wa kujifungua. Vifo vingi vya watoto wachanga wanaoshindwa kupumua mara baada ya kuzaliwa vinatokana na kuwa watoa huduma wengi hawana stadi za kutosha kumsaidia mtoto kupumua.

(Training on how to help the baby breathe is very important for each provider who cares for pregnant mothers during childbirth. Most newborn deaths due to birth asphyxia are due to lack of sufficient skills among many providers to help the baby breathe.)

Mtoa huduma uliyepata mafunzo ya siku moja ya jinsi ya kumsaidia mtoto mchanga kupumua, unalo jukumu la kuwafundisha watumishi wenzako ambao hawakupata fursa ya kuhudhuria mafunzo haya, hususan watumishi walio katika wodi ya kujifungulia na chumba cha upasuaji ili waweze kupata ujuzi na stadi za kutoa huduma hii muhimu na ya dharura.

(As a provider who received a one-day training on how to help the baby breathe, you have the responsibility of teaching your colleagues who have not had the opportunity to attend this training -- particularly those working in the labour ward and the operating theatre – o they can acquire the same knowledge and skills to provide this important and emergency care.)

Wafundishe watoa huduma wenzako kwa kufuata mtiririko wa mafunzo haya kama inavyoelekezwa katika mpango kazi na kwenye kitabu cha mafunzo ya jinsi ya kumsaidia mtoto mchanga kupumua. Mafunzo haya yafanyike kwa vitendo kwa kutumia mdoli. Masomo makuu manne ya mafunzo haya yameainishwa katika mpango kazi kama ifuatavyo:

(Train your fellow healthcare providers to follow the flow of this training as described in the action plan as well as in the training book on how to help the baby breathe. This training should be done in practice by using a mannikin. Four main subjects of this study have been identified in the action plan as follows:)

1. Maandalizi kabla ya kumsaidia mama kujifungua *(Prepare for birth)*
2. Kutoa huduma muhimu kwa mtoto mchanga *(Provide essential care for the newborn)*
3. Kumsaidia mtoto mchanga kupumua ndani ya dakika moja ya dhahabu *(Help the newborn breathe within the Golden Minute)*
4. Kuboresha upumuaji *(Improve ventilation)*

1. **MAANDALIZI KABLA YA KUMSAIDIA MAMA KUJIFUNGUA *(Prepare for birth)***

Hatua ya kwanza: Anza kwa kuchunguza hali halisi ya utoaji huduma kwa mama wakati wa kujifungua na kwa mtoto mchanga atakayezaliwa. Chunguza kama watoa huduma katika wodi ya kujifungulia wanafanya maandalizi ya kabla ya kujifungua kama muongozo unavyoelekeza. Baini mapungufu yaliyopo.

*(**Step One:** Start by exploring the conditions of care for a mother during delivery and for the baby that will be born. Find out if service providers in the labour and delivery ward have made the needed child birth preparations as per guidelines. Take note of the gaps.)*

Hatua ya Pili: Toa mafunzo kwa kuonyesha kwa vitendo jinsi ya kufanya maandalizi kabla ya kumsaidia mama kujifungua. Waelekeze watoa huduma wenzako jinsi ya kufanya maandalizi sahihi kabla ya kumsaidia mama kujifungua (rejea kitabu cha mafunzo uk. 8). Kisha wape fursa kila mmoja kufanya mazoezi ukiweka msisitizo kurekebisha mapungufu yaliyojitokeza kwenye uchunguzi wako hapo juu.

*(**Step Two:** Facilitate a practice on how to make preparations for assisting a mother during child birth. Demonstrate to your colleagues how service providers should make appropriate preparations to help a mother during delivery (see the provider guide p. 8). Then give everyone an opportunity to practice while putting more emphasis on corrective actions based on gaps identified above.)*

Vidokezo vya maandalizi kabla ya kujifungua ni kama ifuatavyo: *(Steps to take prior to delivery of the newborn:)*

- **Kuandaa msaidizi na mpango wa dharura**: Toa maelekezo kwa msaidizi afanye nini endapo dharura itatokea -- nani wa kuitwa kwa msaada zaidi, mawasiliano, usafiri na huduma ya rufaa (rejea uk 6).
  *(**Identify the helper and review the emergency plan:** Give instructions to a companion on what to do when an emergency happens -- communication, transport, and referral (refer to page 6).*

- **Kuandaa eneo la kujifungulia**: Eneo liwe safi, lina joto na mwanga wa kutosha.

  ***(Prepare the area for delivery:** The area should be warm, well-lighted, and clean.)*
- **Kuandaa eneo la kumsaidia mtoto kupumua**: Eneo liwe safi na kavu, meza iliyo bapa na godoro, joto na mwanga wa kutosha.

  ***(Prepare an area for ventilation:** The area should be warm, dry, flat, and safe, without risk for falls or other injury and enough light.)*
- **Kunawa mikono**: kwa sabuni na maji safi yanayotiririka au kutumia alcohol. Elezea faida za kunawa mikono: kunazuia kueneza magonjwa yanayoambukiza. Kunawa mikono ni kabla na baada ya kumhudumia mama au mtoto (rejea uk 39).

  ***(Wash hands:** Use clean water with soap or use alcohol to wash hands. Explain that hand washing helps prevent infection (refer to page 39).)*
- **Kuandaa vifaa:** Andaa vifaa na kuhakiki kama vinafanya kazi. Vifaa muhimu ni; glavu pea 3, towel 2, cord ties, mikasi 2, kofia, soksi, saa na vifaa vya kumsaidia mtoto kupumua (suction device, ambu bag/face masks) na stethoscope. Waonyeshe jinsi ya kuhakiki kama bag na mask vinafanya kazi (rejea picha uk 8). Face mask zinazohitajika ni saizi namba 1 kwa mtoto aliyetimia siku, na saizi namba 0 kwa mtoto njiti. Vifaa vyote vinapaswa kutakaswa baada ya kutumia.

  ***(Prepare the equipment:** Prepare and test the equipment to see if they are working. Equipment required are: 3 pairs of gloves, 2 towels, cord ties, 2 scissors, cap/head cover, stocking to cover the legs, timer (watch/clock), equipment for newborn resuscitation (suction device, ambubag/face masks), and stethoscope. Demonstrate how to test the equipment. Face masks of size 0 for a preterm baby and size 1 for a term baby are required.)*

Kumbuka: Fanya tena uchunguzi wa hali halisi ya maandalizi kabla ya kumsaidia mama kujifungua kwa kila shifti kuona kama watoa huduma wameelewa na wanazingatia na kutekeleza kama ulivyowafundisha.

***(Note:** Make more observations of the actual practice of preparations before assisting mothers giving birth in each shift of providers to assess if they have understood and adhere to what you trained them on.)*

2. KUTOA HUDUMA MUHIMU KWA MTOTO MCHANGA *(ESSENTIAL NEWBORN CARE)*

Hatua ya kwanza: Anza kwa kufanya uchunguzi wa jinsi watoa huduma wanavyozalisha na kutoa huduma muhimu kwa mtoto mchanga asiye na tatizo la kupumua. Ainisha mapungufu yatakayojitokeza.

(Step one: observe the providers on how they conduct deliveries and provide essential newborn care for newborns who breathe normally. Identify the gaps.)

Hatua ya pili: Toa mafunzo kwa kuonyesha kwa vitendo jinsi ya kutoa huduma muhimu kwa mtoto mchanga (rejea uk. 10-15). Kisha wape fursa kila mmoja kufanya mazoezi ukiweka msisitizo kurekebisha mapungufu yaliyojitokeza kwenye uchunguzi wako hapo juu. Waelekeze na kuwaonyesha kwa vitendo jinsi ya kutoa huduma muhimu kwa mtoto mchanga kufuatana na muongozo ulio kwenye mpango kazi (kwa kutumia mtoto aliyezaliwa labour ward au mdoli). Kisha wape nafasi ya kufanya mazoezi kwa vitendo wawiliwawili kwa kutumia mdoli.

(Facilitate practice on how to provide essential newborn care for every baby (refer to pages 10-15). Give an opportunity to every provider to practice while emphasizing correcting the observed gaps in practice as observed in step one above. Explain and demonstrate to providers how to provide essential newborn care according to protocol/action plan (using mannikin or newborn recently born in the labour ward). Then give them an opportunity to practice in pairs of two providers using simulators.)

Vigezo vya jinsi ya kutoa huduma muhimu kwa mtoto mchanga *(Steps for providing essential newborn care)*

- **Weka kitambaa (towel) kwenye tumbo la mama**: Kitambaa kinawekwa kabla mtoto hajazaliwa-hii inarahisisha kumkausha mtoto na kumweka mama katika hali ya ukavu. Unapoondoa kitambaa kilicholoana mtoto anakuwa ngozi kwa ngozi na mama.
  (Put a clean dry towel/drape on the mother's abdomen. This towel will be used to dry the baby.)

- **Futa mtoto mdomo, pua na macho:** Tumia sterile gauze (gauze moja kwa kila sehemu). Onesha namna ya kumfuta mtoto kwa kuanzia mdomo, pua, kisha macho ukitumia gauze moja kwa jicho moja.
  (Wipe the mouth, nose, and eyes using sterile gauze (one piece of gauze for each part). Demonstrate how to wipe the baby starting from the mouth then nose and eyes using separate pieces of gauze in each eye.)
- Zingatia hatua za uzalishaji sahihi, angalia muda aliozaliwa mtoto.
  (Follow the procedure for conducting a delivery and note time of birth.)
- **Chunguza kama mtoto anapumua vizuri.**
  (Check if the baby breathes normally.)
- **Muweke mtoto tumboni kwa mama juu ya kitambaa.** Rejea picha uk 10.
  (Put the baby on mother's abdomen with the dry towel (refer to page 10).)
- **Mkaushe mtoto vizuri**: Kukausha kunasaidia kumpa mtoto joto na kuchochea kupumua. Mkaushe mtoto vizuri ukianzia na kichwa, mwili, mikono, na miguu ukizingatia kwenye mikunjo ya mwili kama shingo, kwapa, mapaja nk (rejea uk 10). Kama mtoto analia, inaashiria kuwa anapumua na hahitaji msaada wa kupumua (rejea uk 11).
  ***(Dry the baby well**. Drying helps keep the baby warm and stimulates breathing. Dry the baby well from head, body, hands, legs, flexion areas of neck, axilla, and thighs (refer to page 10). If the baby cries, it shows that it is breathing normally and does not need help to breathe.)*
- **Mpatie mtoto joto**: Toa kitambaa kilicholoa, muweke mtoto ngozi kwa ngozi kwenye kifua cha mama, mfunike mtoto kwa nguo/khanga kavu, mvalishe kofia na soksi (rejea uk 12).
  ***(Remove the wet towel and put the baby skin-to-skin** on the mother's abdomen and cover the baby with a dry cloth, put cap on the head, and cover the legs with stocking.)*
- Endelea kuangalia kama mtoto anapumua vizuri.
  (Continue observing if the baby is breathing well.)
- **Kata kitovu**: Badilisha glavu, funga na kata kitovu kama muongozo unavyoelekeza (rejea uk 13).
  ***(Cutting the cord**: change the gloves and cut the cord as described in the guidelines (refer to page 13).)*
- **Unyonyeshaji:** Hamasisha unyonyeshaji ndani ya saa 1 baada ya kuzaliwa.
  ***(Breast feeding:** encourage early breast feeding within one hour of birth.)*

- **Utunzaji wa macho**-Mpatie dawa ya macho-Tetracycline eye ointment 1%.
  *(**Eye care**: put tetracycline eye ointment 1% on both eyes.)*
- Mueleekeze mama jinsi ya kutunza kitovu, kuendelea kunyonyesha, kutunza joto. Mtoto aogeshwe baada saa 24 za kuzaliwa ili kuepuka baridi ambayo ni hatari kwa mtoto mchanga.
  (Instruct the mother on how to provide clean cord care, continue breastfeeding, and keep the baby warm. Tell the mother that she should delay bathing the newborn until 24 hours after birth to avoid the baby getting cold/hypothermic, which is dangerous for the baby.)
- Wakati wote mtoa huduma anatakiwa kuangalia kama mtoto anapumua vizuri.
  (A provider should continue to observe the baby and ensure she breathes well.)

Kumbuka: Fanya tena uchunguzi wa hali halisi ya huduma muhimu kwa mtoto mchanga kwa kila shifti kuona kama watoa huduma wameelewa na wanatekeleza hatua zote zilizoainishwa hapo juu.

*(**Note:** Repeat the observations of the actual state of pre-girth preparations to ensure providers on each shift understand and adhere to what was taught.)*

3. KUMSAIDIA MTOTO KUPUMUA NDANI YA DAKIKA MOJA YA DHAHABU *(HELPING THE BABY BREATHE WITHIN THE GOLDEN MINUTE)*

Hatua ya kwanza: Anza kwa kuchunguza huduma za kumsaidia mtoto mchanga kupumua ndani ya dakika moja katika kituo chako ili kubaini mapungufu yaliyopo.

(Step one: observe the current services for helping babies breathe in the health facility and identify the gaps.)

Hatua ya pili: Toa mafunzo na kuonyesha kwa vitendo jinsi ya kumsaidia mtoto kupumua (rejea uk. 18-21). Kisha wape fursa kila mmoja kufanya mazoezi ukiweka msisitizo kurekebisha mapungufu yaliyojitokeza kwenye uchunguzi wako hapo juu. Waelekeze na kuwaonyesha kwa vitendo stadi za kumsaidia mtoto kupumua huduma ndani ya dakika moja kwa au mdoli. Kisha wape nafasi ya kufanya mazoezi kwa vitendo wawiliwawili kwa kutumia mdoli.

(Facilitate practice on how to help a baby breathe (refer to pages 18-21). Give an opportunity to providers to practice while emphasizing correcting the observed gaps in practice as seen in step one above. Explain and demonstrate the skills on how to help a baby breathe within one minute using a mannikin. Let the providers practice in pairs).

Vigezo vya jinsi ya kumsaidia mtoto mchanga kupumua: ***(Steps for helping the baby breathe:)***

- **Weka kitambaa (towel) kwenye tumbo la mama.**
  (Put a dry towel on the mother's abdomen.)
- **Futa mtoto mdomo, pua na macho.**
  (Wipe the mouth, nose, and eyes.)
- **Zingatia hatua za uzalishaji sahihi, angalia muda aliozaliwa mtoto.**
  (Follow the procedure for conducting a delivery and note the time of birth.)
- **Muweke mtoto tumboni kwa mama juu ya kitambaa.**
  (Put the baby on the mother's abdomen.)
- **Mkaushe mtoto vizuri.**
  (Dry the baby well.)
- **Mpatie mtoto joto**: Toa kitambaa kilicholoa, muweke mtoto ngozi kwa ngozi kwenye kifua cha mama, mfunike mtoto kwa nguo/khanga kavu.
  ***(Keep the baby warm**: Remove the wet towel and put the baby skin-to-skin on the mother's chest and cover the baby with a dry cloth.)*
- **Tambua mtoto halii/hapumui**: Mfanyie tathmini mtoto na ujiulize, je mtoto analia? Kama halii/hapumui msaidie kupumua ndani ya dakika moja.
  ***(Check if the baby is crying/breathing**: Quickly evaluate if the baby is crying. If the baby is not crying/not breathing, then quickly help the baby breathe within the Golden Minute.)*
- **Safisha njia ya hewa:** Mlaze mtoto ubavu huku shingo yake ikiwa imenyooka kidogo kusaidia kuweka njia ya hewa wazi. Safisha kuanzia mdomoni halafu puani. Chunguza upumuaji (rejea uk 16).
  ***(Clear the airway:** position the head slightly extended, remove secretions from the airway if they are blocking the mouth or nose. Check beathing (refer to page 16).)*
- **Mshtue apumue**: Mshtue kwa kumsugua mgongoni mara moja au mbili. Usichelewe au kutumia muda mrefu kushtua kupumua.

*(**Stimulate breathing**: Rub the back 1 or 2 times. Don't delay or take a long time to stimulate breathing.)*

- **Chunguza upumuaji**: Kwa kujiuliza je mtoto anapumua vizuri. Kama mtoto anapumua vizuri endelea kumpatia huduma muhimu (Rangi ya kijani-mpango kazi). Kama hapumui.

  *(**Check breathing**: Is the baby breathing well? If the baby is breathing well, continue with essential care (green colour on the action plan).)*
- **Kata kitovu**: Vua glavu ya juu, funga na kukata kitovu haraka kwa kukadiria urefu.

  (If the baby is not breathing, cut the cord immediately.)
- Mpeleke mtoto kwenye eneo la kumpatia pumzi, simama nyuma ya kichwa cha mtoto na chagua mask sahihi.

  (Take the baby to the resuscitation area, select the appropriate mask.)
- **Mpatie mtoto pumzi**: Mlaze mtoto chali, ukiwa umesimama upande wenye kichwa cha mtoto. Hakikisha shingo ya mtoto imenyooka kidogo (neutral position). Weka mask kwenye uso na ifunike pua na mdomo, kisha minya bag taratibu ili kumpa pumzi 40-50 kwa dakika (rejea uk 20-21).

  *(**Ventilate the baby**: ensure the neck is slightly extended, put the mask covering the nose and mouth then ventilate at a rate of 40-50 per minute (refer to pages 20-21).)*
- **Endelea kumpatia mtoto joto:** Hakikisha mtoto amefunikwa vizuri na kuacha kifua wazi kwa ajili ya kuangalia upumuaji wakati wa kumpatia pumzi.

  *(**Keeping baby warm:** ensure the baby is well covered, leave chest open to observe the breathing.)*
- **Tambua anapumua vizuri:** mtoto anayepumua vizuri analia au anapumua taratibu/kimya na kifua kinatanuka kawaida na rangi ya mtoto inakuwa ya pinki. Kama anapumua vizuri endelea kumpatia huduma muhimu (rangi ya kijani-mpango kazi).

  (A baby who is breathing well, crying or breathing quietly regularly and chest is moving with breathing and has pink colour. If the baby is breathing well continue to provide essential care (yellow colour in the action plan).)

Kumbuka: Fanya tena uchunguzi wa hali halisi ya huduma ya kumsaidia mtoto kupumua ndani ya dakika moja kwa kila shifti kuona kama watoa huduma wameelewa na wanatekeleza kama ulivyowafundisha.

***(Note:** Each shift, check that service providers have understood and implement helping baby breathe within the golden minute as it has been taught.)*

4) **JINSI YA KUBORESHA UPUMUAJI** ***(How to improve ventilation)***

Endapo mtoto hatapumua baada ya dakika moja ya dhahabu, omba msaada, na boresha upumuaji kwa kufuata hatua zifuatazo: *(If the baby is not breathing after the Golden Minute, call for help and improve breathing as follows:)*

- Angalia mdomo, nyuma ya koo,na kwenye pua kama kuna mchojozo.
  (Look if there are secretions in the mouth, pharynx, and nose.)
- Weka kichwa upande na safisha njia ya hewa kwa kutumia kifyonza mchojozo kama inavyohitajika.
  (Remove the secretions from air ways by using suction device as needed.)
- Weka kichwa cha mtoto vizuri shingo ikiwa imenyooka kidogo.
  (Ensure the neck is slightly extended.)
- Fungua mdomo wa mtoto kidogo weka tena mask kwenye uso ishike kabisa bila ya hewa kupenya pembeni.
  (Open the mouth slightly and put the mask again and ensure there is no leakage.)
- Minya bag kwa nguvu ili kutoa pumzi kubwa zaidi, endelea kutoa pumzi chunguza mapigo ya moyo kila baada ya dakika moja (rejea uk 26-27).
  (Ventilate with bag and mask and check the heart rate every one minute (refer to pages 26-27).)
- Tambua bado hapumui vizuri: kama mtoto hapumui vizuri baada ya kumpa pumzi vizuri zaidi mfanyie tathimini kwa kujiuliza je mapigo ya moyo ni ya kawaida au taratibu (rejea picha uk 27).
  (If the baby is still not breathing after ventilation, check if hert rate are normal or slow (refer to page 27).)
- Tambua mapigo ya moyo ni ya kawaida au taratibu: Mapigo ya moyo ya kasi ya 100 au zaidi kwa dakika moja ni ya kawaida, na mapigo ya moyo

ya kasi ya chini ya 100 kwa dakika moja ni ya taratibu, endelea kumpatia pumzi na tafuta ushauri wa kitaalamu au rufaa.
(Heart rate of 100 or more beats per minute is normal, and below 100 beats per minute is abnormal. Continue to ventilate and seek advanced care.)

- Baada ya dakika kumi zakumpatia pumzi, mtoto hapumui na hamna mapigo ya moyo, acha kumpa pumzi, mtoto amefariki.
  (After ten minutes of ventilation, and the baby is not breathing, no heart rate, consider stopping ventilation, counsel the care givers/mother.)

ZINGATIA

Iwapo kuna mekonium safisha njia ya hewa kabla ya kumkausha mtoto. Mfuatilie kwa karibu mtoto aliyesaidiwa kupumua akiwa na mama kwa kufuatilia viashiria vifuatavyo ; upumuaji, rangi, mapigo ya moyo, joto, uchangamfu na unyonyaji wa mtoto (rejea uk 41).

(If there is meconium, clear the airway. Observe closely the newborn and monitor breathing, skin clour, heart rate, temperature, activity, and breastfeeding (refer to page 41).)

OSCES za kufanya mazoezi/kujifunzia kazini: ***(OSCEs to practice/learn on the job:)***

Maelekezo kwa mwezeshaji: Soma kwa sauti kwa mshiriki maelekezo yafuatayo. Kwa kila hatua weka maksi "1 au 3" kama hatua imefuatwa na weka "0" kama hatua haikufuatwa. Maksi zinazohusika kwa kila hatua zimeoneshwa kwenye safu ya mwisho kulia; kisha andika Jumla kuu. Onyesha au sema vitu vitakavyompa vidokezo mtu anayefanya mazoezi, kwa mfano "imetokea mtoto bado hajalia utafanya nini?"

(Facilitator's note: read the scenario and in each step mark 1 or 3 if the step was correctly performed by the provider and 0 if not correctly performed. The marks for each step are shown in the far right column. Provide prompts by saying or showing the action, for example, ''What would you do if the baby is not crying.'')

MFANO WA 1: KISA MKASA 1 CASE SCENARION 1 *(Case scenario 1)*

"Nitasoma mfano wa historia ya mtoto anayezaliwa. Tafadhali sikiliza kwa makini halafu uoneshe hatua utakazochukua kwa vitendo. Sitakupa mrejesho hadi mwisho wa zoezi hili"

("I am going to read a role play case. Please listen carefully, and then show me the actions you would take. I will indicate the baby's responses, but I will provide no other feedback until the end of the case.")

(MAANDALIZI KABLA YA KUJIFUNGUA NA HUDUMA MUHIMU KWA MTOTO
(Preparation for birth and essential newborn care)

		Weka alama stahiki	Alama inayohitajika
**Zingatia: Kituo cha OSCE kina alcohol handrub au sinki lililochorwa kwenye karatasi (*Note: the OSCE are should include alcohol rub or drawing of a sink)*** **Mimba iliyotimia umri; mtoto atazaliwa ndani ya dakika tano zijazo; hakuna mekonium, utafanya maandalizi gani kabla ya kuzalisha?** *(You are called to assist the delivery of a term baby. There are no complications in the pregnancy. The baby will be born within 5 minutes. What will you do to prepare before birth?)*			

MAANDALIZI KABLA YA KUJIFUNGUA			
1	*Anaandaa msaidizi*<br>*Anaandaa eneo la kuzalishia (liwe: safi, lenye hewa, joto na mwangawa kutosha)*<br>*(Identifies a helper and reviews an emergency plan; prepares the area for delivery (warm, well-lighted, clean))*		1
2	*Ananawa mikono au kutumia alcohol handrub*, anavaa *gloves pea 2*<br>*(Washes hands using alcohol handrub, wears 2 pairs of gloves)*		1
3	*Anaandaa sehemu ya kumsaidia mtoto kupumua anakagua vifaa na kuangalia kama vinafanya kazi*<br>*(Prepares a resuscitation area and checks the function of the bag, mask, and suction devices)*		1
**HUDUMA MUHIMU KWA MTOTO** ***(Essential newborn care)***			
Dodosa: Baada ya dakika mbili mpe mdoli mshiriki na sema,<br>***"Unazalisha mtoto, maji ya amnion ni safi. sasa amezaliwa. Onesha jinsi utakavyomhudumia mtoto."***<br><br>***(Prompt: after 2 minutes give the NeoNatalie to the provider and say, "You have delivered a baby, the mother's amniotic fluid is clear. Demonstrate how you will care for the baby.")***			
4	*Anamweka mtoto kifuani kwa mama na kumweleza mama jinsia ya mtoto na hali ya mtoto kuwa ni nzuri*<br>*(Puts the baby onto mother's chest, and informs the mother the sex of the child and that the child is doing well)*		1
5	*Anamkausha mtoto vizuri (akianzia na kichwa, mwili, mikono, na miguu akizingatia kwenye mikunjo ya mwili kama shingo, kwapa, mapaja nk)*<br>*(Dries the baby well (starting from the head, body, hands, and legs paying attention to body parts with folds))*		3

6	*Anabadili nguo iliyolowa kwa mtoto na kumfunika kwa nguo iliyo kavu*<br>*(Changes the wet towel and covers the baby with a dry cloth)*		1
7	*Anampa mtoto joto (anamweka mtoto ngozi kwa ngozi na mama, anamfunika na nguo kavu na kumvalisha kofia na soksi.)*<br>*(Keeps the baby warm (puts the baby skin-to-skin and covers her with dry clothes including the head and feet))*		1
8	Anaangalia mtoto anavyopumua<br>*(Observes the breathing of the baby)*		1
9	*Anabadilisha glavu na kuvaa nyingine*<br>*(Changes gloves)*		1
10	*Anafunga na kukata kitovu kwa usahihi*<br>*(Correctly cuts the cord)*		1
11	*Anahamasisha unyonyeshaji*<br>*(Encourages early breast feeding)*		1
12	*Anamwekea mtoto dawa ya macho*<br>*(Applies baby's eye ointment)*		1
	Jumla kuu (Huduma muhimu kwa mtoto mchanga)<br>***Total score (Essential newborn care)***		
**Toa mrejesho ukianza na hatua zilizofanywa vizuri; kisha taja na kurudia kuonesha hatua zilizofanywa isivyosahihi; ziandike ukisisitiza ziendelee kufanyiwa mazoezi kazini**<br>***(Give feedback starting with well performed steps and then emphasize areas for improvement)***			

Maelekezo kwa mwezeshaji: Mwelekeze anayefanya mazoezi

(Instructions to a facilitator: Guide a provider to practice)

Fikiria ulishafanya maandalizi yote kabla ya kuzalisha mtoto; umenawa; na kuvaa glavu mbili tayari.

(Assume you have already done all the preparations needed before attending child birth, washed your hands, and wore 2 pairs of gloves.)

MFANO 2: KISA MKASA 2

(Case scenario no. 2)

"Nitasoma mfano wa historia ya mtoto anayezaliwa, ambaye halii na anahitaji msaada wa kupumua.Tafadhali sikiliza kwa makini halafu uonyeshe hatua utakazochukua kwa vitendo. Nitaonyesha jinsi mtoto atakavyoitikia kwa kutumia mdoli (au maneno), lakini sitakupa mrejesho hadi mwisho wa zoezi hili"

("I'm going to read a story of a baby born who is not crying and needs help to breathe. Please listen carefully, and then show me the actions you would take. I will indicate the baby's responses, but I will provide no other feedback until the end of the case.")

DAKIKA YA DHAHABU, KUBORESHA UPUMUAJI NA MPANGO WA RUFAA

(Golden Minute, improving breathing, and referral plan)

		Weka alama stahiki	Alama inayohitajika
Dodosa: "Umezalisha mtoto mimba imetimia umri wake*. Maji ya amnion ni safi, mtoto halii na hajapumua. Utafanya nini kwa haraka na usahihi kumsaidia mtoto?"** ***(Prompt: "You delivered a full-term baby, clear amniotic fluid, not crying or breathing. What will you quickly do to help the baby breathe?")			
1	*Anamkausha mtoto vizuri*<br>*(Dries the baby well)*		3
2	*Anabadili nguo iliyolowa kwa mtoto na kumfunika kwa nguo iliyo kavu*		1

	(Changes the wet towel and covers the baby with a dry cloth)		
3	*Anaweka kichwa upande na anasafisha njia ya hewa kwa kutumia kifaa cha kufyonza*<br>*(Positions the head in a slightly extended position and clears the airway)*		3
4	*Anatumia kifyonza mchojozo kwa usahihi (anaminya ikiwa nje na kuachia anapoingiza mdomoni au puani)*<br>*(Uses the penguin sucker correctly (presses the penguin sucker while outside and releases when inside the mouth or nose))*		1
5	*Anaanza kusafisha njia ya hewa kwa kuanzia mdomoni anafuatia puani*<br>*(Clears the airway by suctioning the mouth first and then the nose)*		1
6	*Anamshtua apumue* kwa kumsugua mgongoni mara moja au mbili<br>*(Stimulates breathing by rubbing the back 1 or 2 times)*		3
Dodosa:* Mtoto bado hapumui utafanya nini?**<br>(Prompt: if the baby is still not breathing, what will you do?)***			
7	*Anavua glavu*<br>*(Changes gloves)*		1
8	*Anabana kitovu kwa fosepsi na Anakata kitovu*<br>*(Cuts the cord)*		1
9	*Anamuweka mtoto vizuri sehemu safi, bapa, kavu,yenye joto na mwanga na anasimama upande wa kichwa cha mtoto ili aweze kumsaidia kupumua*<br>*(Places the baby in an area that is clean, flat, warm, with enough light, and prepares to help the baby breathe)*		1
10	*Anampa mtoto joto wakati wa kumsaidia kupumua*<br>*(Keeps the baby warm during ventilation)*		1

11	*Anachagua mask yenye ukubwa sahihi na kuanza kumpa mtoto pumzi ndani ya dakika moja*<br>*(Selects a mask of appropriate size and starts ventilating the baby within one minute)*		1
12	*Anaminya bag kupanua kifua taratibu kwa kasi ya pumzi 40-50 kwa dakika*<br>*(Ventilates with the bag and mask at a rate of 40-50 per minute)*		1
Dodosa; Dakika moja imeisha lakini mtoto bado hapumui,utafanya nini ili kuboresha utoaji wa pumzi<br>***Prompt: baby still not breathing after one minute, what will you do?***			
13	*Anaomba msaada*<br>*(Calls for help)*		1
14	*Anaangalia mdomo,nyuma ya koo,na kwenye pua kama kuna mchojozo*<br>*(Checks for secretions in the mouth, pharynx, and nose)*		1
15	*Anaweka kichwa upande na anasafisha njia ya hewa kwa kutumia kifyonza mchojozo kama inavyohitajika*<br>*(Clears the airway using the suction device as needed)*		1
16	*Anaweka kichwa cha mtoto vizuri shingo ikiwa imenyooka Kidogo*<br>*(Positions the head with the neck slightly extended)*		1
17	*Anafungua mdomo wa mtoto kidogo, anaweka tena mask kwenye uso ishike kabisa bila hewa kupenya pemebeni*<br>*(Applies the mask and ensures no air leakage)*		1
18	*Anaminya bag kwa nguvu ili kutoa pumzi kubwa zaidi*<br>*(Ventilates with the bag and mask with higher pressure)*		1
19	*Anaendelea kutoa pumzi na anakagua (anahesabu) mapigo ya moyo kila baada ya dakika moja*		1

	(Continues with ventilation and checks the heart rate every minute)		
**Dodosa: Mtoto hapumui na mapigo ya moyo ni taratibu chini ya 100 kwa dakika**<br>***(Prompt: a baby is not breathing and the heart rate is slow less than 100 beats per minutes)***			
20	*Anaendelea kumpa pumzi kwa dakika 10-20 (akikagua mapigo ya moyo kila baada ya dakika 1) na kumpa rufaa kwa msaada wa utaalamu zaidi*<br>*(Continues ventilation for 10-20 minutes (while checking the heart rate every minute) and makes a referral for advanced care)*		1
21	*Anatambua kwamba mtoto anatakiwa aendelee kupewa pumzi bila kuacha wakati wa kumsafirisha kwa rufaa*<br>*(Recognizes that the baby needs continued ventilation during referral)*		1
	**JUMLA KUU**<br>***Total score***		
**Toa mrejesho ukianza na hatua zilizofanywa vizuri; kisha taja na kurudia kuonesha hatua zilizofanywa isivyosahihi; ziandike ukisisitiza ziendelee kufanyiwa mazoezi kazini**<br>***(Give feedback starting with well performed steps and then emphasize areas for improvement)***			
